# Spectral tuning of Bloch Surface Wave resonances by light-controlled optical anisotropy

**DOI:** 10.1515/nanoph-2022-0609

**Published:** 2023-02-20

**Authors:** Niccolò Marcucci, Maria Caterina Giordano, Giorgio Zambito, Adriano Troia, Francesco Buatier de Mongeot, Emiliano Descrovi

**Affiliations:** Dipartimento di Scienza Applicata e Tecnologia, Politecnico di Torino, Corso Duca degli Abruzzi 24, Torino, 10129, Italy; Dipartimento di Fisica, Università di Genova, Via Dodecaneso 33, Genova, 16146, Italy; Istituto Nazionale di Ricerca Metrologica (INRiM), Strada delle Cacce 91, Torino 10135, Italy

**Keywords:** azopolymers, birefringence, Bloch surface waves, thermal-scanning probe lithography, tunable nano-photonics

## Abstract

Fostered by the recent advancements in photonic technologies, the need for all-optical dynamic control on complex photonic elements is emerging as more and more relevant, especially in integrated photonics and metasurface-based flat-optics. In this framework, optically-induced anisotropy has been proposed as powerful mean enabling tuning functionalities in several planar architectures. Here, we design and fabricate an anisotropic two-dimensional bull’s eye cavity inscribed within an optically-active polymeric film spun on a one-dimensional photonic crystal sustaining Bloch surface waves (BSW). Thanks to the cavity morphology, two surface resonant modes with substantially orthogonal polarizations can be coupled within the cavity from free-space illumination. We demonstrate that a dynamic control on the resonant mode energies can be easily operated by modulating the orientation of the optically-induced birefringence on the surface, via a polarized external laser beam. Overall, reversible blue- and red-shifts of the resonant BSWs are observed within a spectral range of about 2 nm, with a moderate laser power illumination. The polymeric structure is constituted by a novel blend of an azopolymer and a thermally-sensitive resist, which allows a precise patterning via thermal scanning probe lithography, while providing a significant structural integrity against photo-fluidization or mass-flow effects commonly occurring in irradiated azopolymers. The proposed approach based on tailored birefringence opens up new pathways to finely control the optical coupling of localized surface modes to/from free-space radiation, particularly in hybrid organic–inorganic devices.

## 1 Introduction

Tunable photonics deals with micro and nanostructures able to modify some of their optical functions and properties upon external cues, which are typically fed as control means in active devices or measurand in sensing architectures. In many active devices, changes in the refractive index of constituent materials can be obtained after providing an external luminous trigger [1], often in the form of focused laser beams. Modulation strategies involving all-optical control are particularly advantageous in integrated photonic chips as compared to other approaches (e.g. based on thermo-optical or photo-acoustic effects), since they allow reaching a considerable spatial selectivity and a minimal cross-talk with adjacent circuit parts. With this respect, intensive research efforts are presently directed towards new optical materials, such as transparent conductive oxides [2] and semiconductors [3], with tailored linear and non-linear properties enabling an optical modulation based on sufficiently strong, yet reversible, refractive index changes. Alternatively, polymeric compounds containing light-active units (e.g. azobenzenes [4] and dithienylethene [5]) represent a valuable option in hybrid organic–inorganic architectures, mainly because of the large variety of different light-responsive mechanisms available [6] and the moderate power required to the external radiation to trigger the photoswitching functionalities. As an example of integrated hybrid photonic device, azobenzene-functionalized silica toroidal resonators have been demonstrated to show a stable, reversible spectral tunability up to about 4 nm at telecom wavelengths, upon illumination with external laser sources in the visible [7]. While light-induced conformational changes in azobenzene molecules have been successfully used to produce refractive index variations [8] cyclic photoisomerization and the subsequent re-orientation of dipole momenta in azobenzene-containing polymeric films is well-known to result also into remarkable birefringence, whose spatial orientation is determined by the polarization state of the illuminating radiation. Such an optically-induced anisotropy offers intriguing opportunities to tune topological features in planar photonic structures, as recently demonstrated for metasurfaces [9, 10].

**Figure 1:**
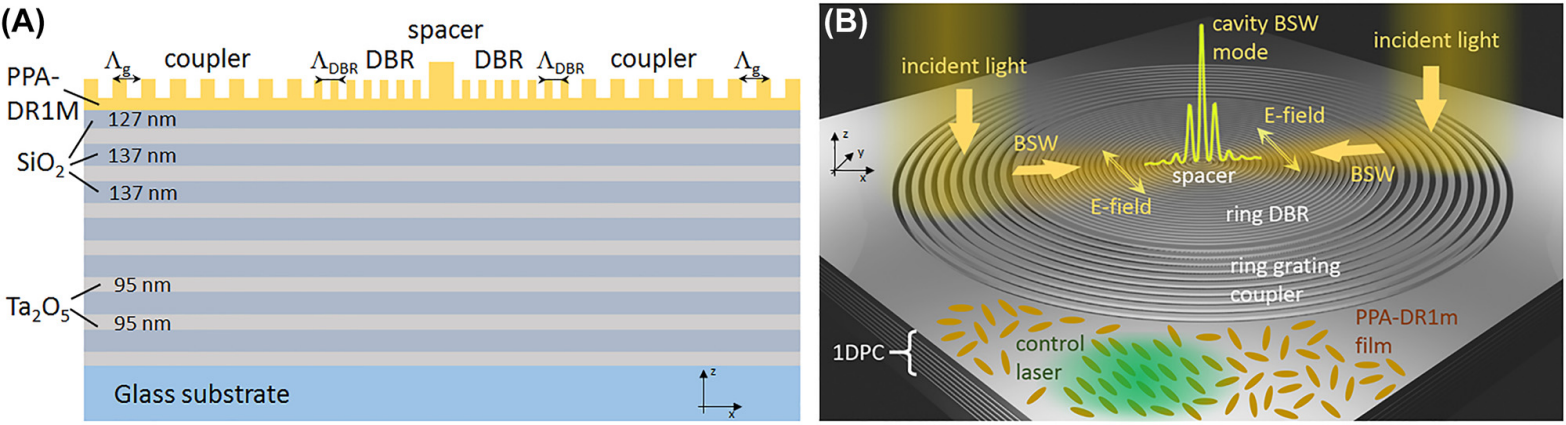
Resonant structure and principle of operation. (A) Vertical cross sectional view of the structure consisting of a dielectric multilayer on glass and a light-responsive polymer structure on top. (B) Illustrative 3D sketch of the resonant 1DPC patterned with the annular grating coupler and the BSW cavity constituted by a distributed Bragg reflector (DBR) surrounding an inner circular spacer. The pattern is inscribed into a 65 nm-thick azopolymeric blend film whose optical anisotropy can be controlled by orienting the alignment of the azobenzene molecules contained therein, via irradiation with an external green laser. The TE-polarized BSW coupling occurs along directions determined by the polarization of the incident white light. If the incident white light is unpolarized, BSW coupling occurs along all radial directions, as allowed by the circular grating. The control laser is typically expanded so that the whole pattern (cavity and grating coupler) is illuminated.

In this work, we present a solid-state dielectric multilayer functionalized with a structured, optically active polymeric film whose birefringence can be controlled via a properly polarized external laser radiation. As a result, we show that the resonances associated to surface modes sustained by the structure can be spectrally modulated over a range of few nanometers in wavelength. The concept is better illustrated in Figure 1. A blend of an optically active azopolymer (DR1M) and a thermally-sensitive resist (PPA) is spun on a dielectric multilayer (one-dimensional photonic crystal -1DPC) to form a very thin film. The 1DPC sustains TE-polarized Bloch surface waves (BSWs) in the visible (details in the Experimental section). A resonant flat-optics nanocavity for BSWs is then fabricated on top of the DR1M-PPA film by means of a novel thermal-scanning probe lithography approach. Thermal-scanning probe lithography (t-SPL) has recently emerged as a very promising approach to locally tailor materials properties at the nanoscale level, thanks to a sharp conductive probe able to induce local and controlled heating of the surface [11]. In this way, high-resolution nanolithography can be performed onto polymeric layers possibly transferred onto other substrates, so that photonic architectures can be engineered to implement various optical functions [12, 13].

An external laser beam with a controlled polarization state produces an optical anisotropy in the DR1M-PPA film that affects the BSW coupling and propagation. Being TE-polarized and mostly confined at the surface, BSWs are better candidates than surface plasmons [14] to sense the in-plane anisotropy [15] inscribed within thin functional azopolymeric films. In the illustrative application reported here and represented in Figure 1, an annular grating coupler and a circular bull’s eye cavity are inscribed within the DR1M-PPA layer, with the twofold goal of coupling BSWs from free-space radiation and resonantly confining them within the cavity inner region. When a linearly polarized control laser (CW doubled-frequency Nd:YAG) forces the azobenzene molecules to orient, thus resulting in birefringence, the BSW cavity modes experience spectral shifts that depend on their polarization state. Such a resonance shift is reversible and can be operated several times without significantly degrading the polymeric pattern, photobleaching effects representing the intrinsic and more critical issue of the proposed tuning mechanism, in terms of repeatability.

## 2 Results and discussion

### 2.1 Bloch surface wave tuning on flat 1DPC

**Figure 2:**
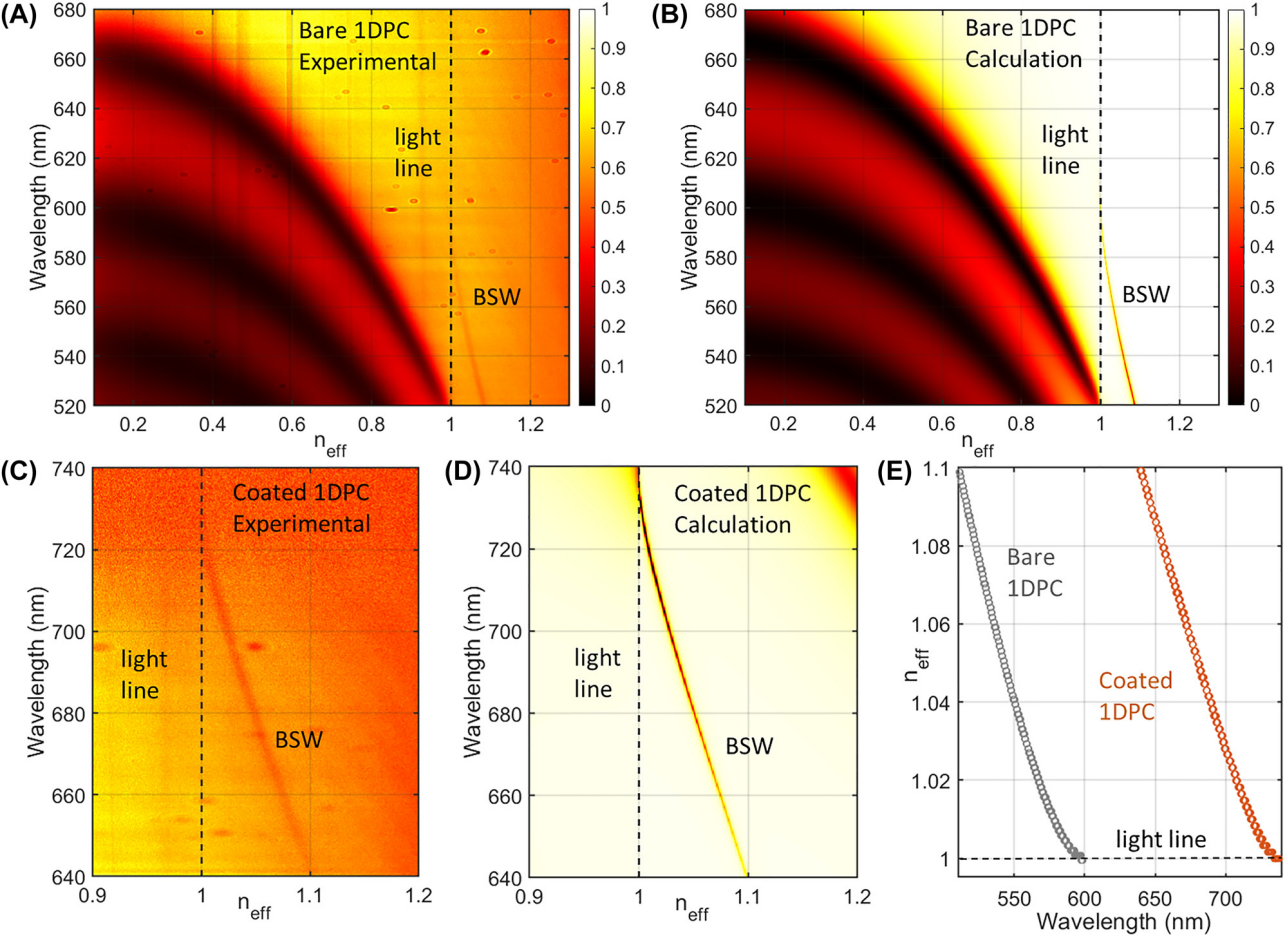
BSW dispersion curves. (A) Measured and (B) calculated angular-resolved spectral reflectivity map of the bare 1DPC; (C) measured and (D) calculated reflectivity maps for the coated 1DPC, where the coating is a 65 nm – thick DR1M-PPA layer with refractive index *n*_p_ = 1.615 ± 0.022 estimated from fitting the experimental data. Note that the halogen lamp spectrum is reaching almost zero intensity at wavelengths above 720 nm. (E) Calculated BSW dispersions for bare and coated 1DPC.

An angularly-resolved spectral reflectivity map is commonly used to investigate the BSW dispersion, with the illumination provided from the glass substrate of the multilayer, through an oil-immersion bulky optics (along the “reflection path”, as described in the Experimental section), which is also used for the collection of the reflected light. The TE-polarized BSW on a bare 1DPC appears as a reflectivity dip located above the light line, as shown in Figure 2A. According to the reference axes of the optical setup, the collected light is polarization-filtered so that the reflectivity map and the corresponding BSW are polarized along the *x*-direction. Calculations performed by means of the transfer matrix method (Figure 2B) are found to agree very well with the experimental data. When the 1DPC is coated with a dielectric thin layer, the BSW dispersion is red-shifted according to the well-known dielectric loading effect [16] (Figure 2C). The extent of the red-shift depends on the thickness and the refractive index of the coating. In this case, the refractive index of the DR1M-PPA blend spun on the 1DPC is unknown but it can be evaluated after quantifying the BSW shift and the coating thickness. An AFM analysis reveals a polymer film as thin as 65 ± 3 nm (details in the Experimental section), while, the calculated BSW dispersion showing the best matching with the experimental data is obtained with a real refractive index *n*_p_ = 1.615 ± 0.022, after several computing iterations of the transfer matrix method (Figure 2D). The estimated refractive index value for the DR1M-PPA blend is in good agreement with a weighted sum of the DR1M and PPA refractive indexes, as seen from previous works [17–19].

**Figure 3:**
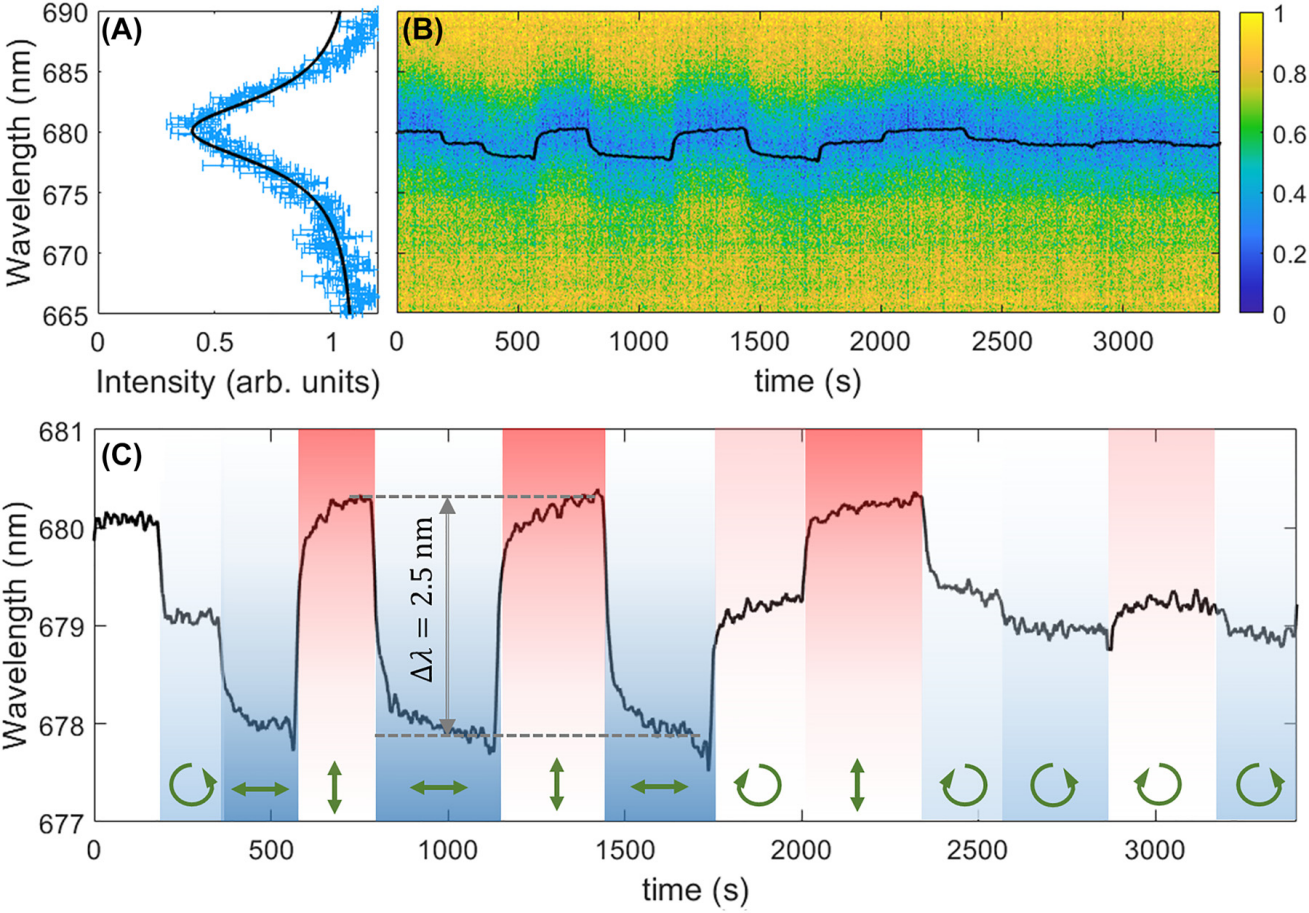
Spectral shift of the BSW resonance. (A) Measured spectral profile of the intensity dip associated to an *x*-polarized BSW at *λ*_0_ = 680 nm. Uncertainties at each wavelength are evaluated by taking the maximum fluctuation of the intensity signal over 5 frame acquisitions (integration time 1 s) in stationary conditions. A Lorentzian fit of the intensity profile is also shown; (B) time evolution of the BSW resonance during laser irradiation; (C) time evolution of the central wavelength of the BSW dip obtained after fitting with a Lorentzian function. Green arrows indicate the polarization state of the illuminating laser: *y*-oriented (vertical), *x*-oriented (horizontal), left- or right-handed. An overall spectral range of about 2.5 nm is observed in BSW tuning.

An expanded laser beam is used to illuminate the full field-of-view of the collecting objective. This is needed in order to obtain a uniform optical anisotropy across the sampled spatial domain that is Fourier-transformed onto the back focal plane (BFP) image fed into the spectrometer through the entrance slit. Measuring birefringence from BFP imaging has been proposed in a recent work involving surface plasmon coupling and detection [20]. The measured laser power hitting the sample is 0.88 mW and the polarization can be changed from linear (either *x*- or *y*-oriented) to circular (either left- or right-handed). The laser spot diameter is 2.5 mm. An initial *x*-polarized BSW resonance peaked at *λ*_0_ = 680 nm (Figure 3A) is monitored over time, as the laser polarization is varied. In Figure 3B the BSW resonance is shown to shift depending on the laser illumination conditions. In order to better evaluate the spectral shift, the BSW dip profile is fitted with a Lorentzian function, whose center wavelength is plotted over time (Figure 3C). At first, the laser is OFF. After about 180 s, the laser is switched ON, in a circular polarization state. The BSW dip is observed to blue-shift by roughly 1 nm, as a consequence of a decrease of the refractive index in the 1DPC plane, sensed by the TE-polarized BSW. This effect results from the azobenzene dipoles driven by the rotating electric field of the laser to orient preferentially out of plane [21]. When the laser polarization is set as linear, for example along the *x*-direction (i.e. parallel to the BSW polarization), the BSW resonance further blue-shifts, as the population of azobenzene molecules oriented along the *x*-axis tends to be depleted. In such a situation, a significant optical anisotropy on the 1DPC plane is produced. As a consequence, when the laser linear polarization is rotated parallel to the *y*-axis, a red-shift of about 2.5 nm is found. Interestingly, the BSW dip position is red-shifted only slightly with respect to the very initial position (laser OFF), when all azobenzene molecules have a uniform orientation in 3D. This can be explained by recalling that a *y*-polarized laser makes the azobenzene dipoles to orient preferentially parallel to the *xz*-plane [21], thus providing a weak increase of the refractive index along the *x*-direction. In this framework, the DR1M-PPA coating behaves as a negative uniaxial crystal having *n*_‖_ < *n*_⊥_ [22] where the extraordinary refractive index *n*_‖_ is parallel to the laser polarization direction and the ordinary refractive index *n*_⊥_ is orthogonal. Overall, the observed BSW shift relies on a birefringence Δ*n ≅* 0.01, which is in agreement with previous works [23] at comparable irradiation intensity levels ( ∼18 mW/cm^2^). The maximum modulation of the BSW resonance is eventually obtained when operating in presence of a significant in-plane birefringence that can be rotated by 90° by changing the linear polarization of the laser from *x*-oriented to *y*-oriented and viceversa.

The red/blue-shift is rather reversible. Interesting to note, the BSW spectral position corresponding to the circular polarization of the laser is roughly located in the middle of the full spectral range of the modulation. However, a closer look reveals that left-handed and right-handed polarizations affect differently the dip position, probably due to some ellipticity of the laser that is not perfectly compensated.

### 2.2 Characterization and modelling of a 2D BSW cavity

**Figure 4:**
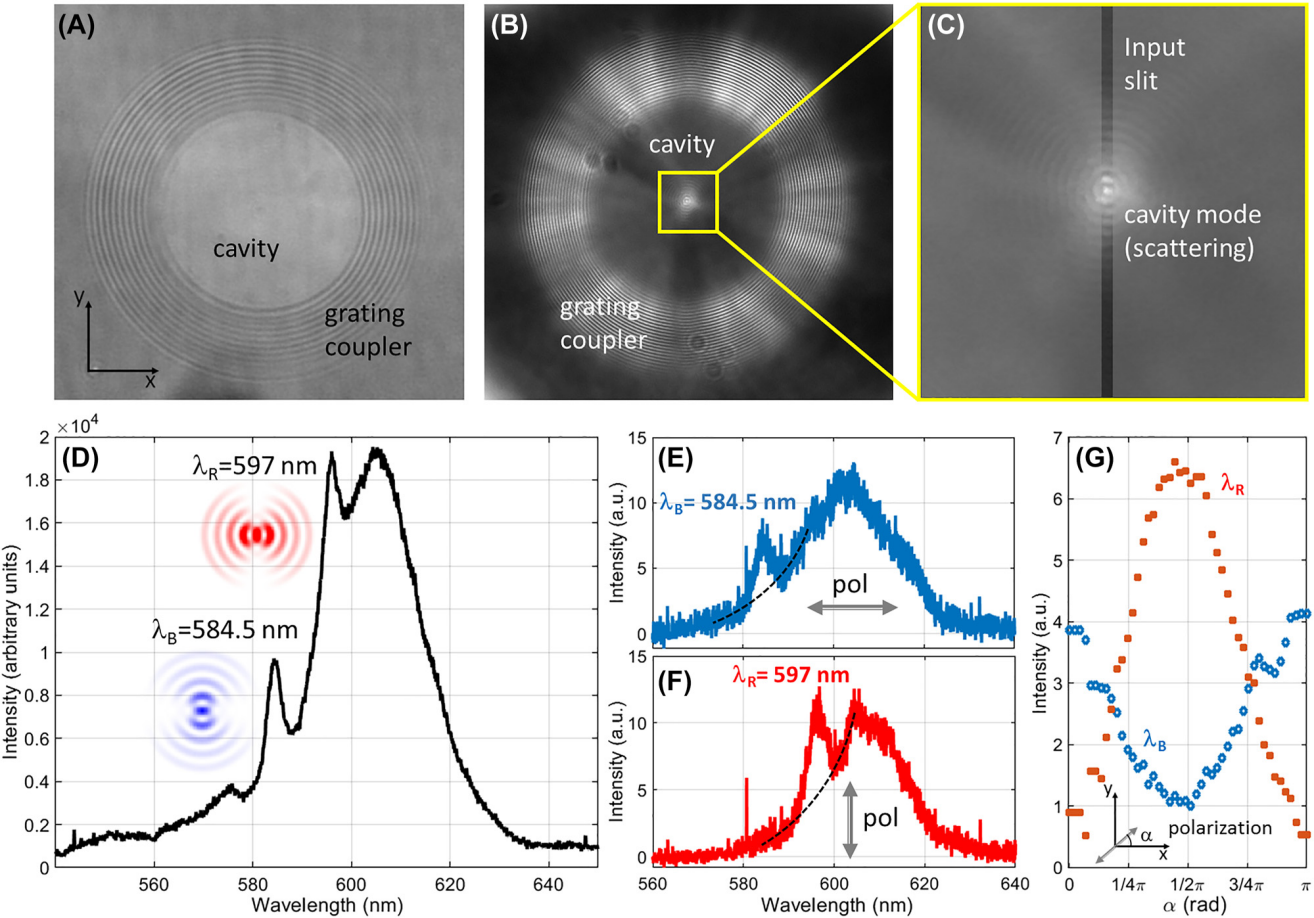
BSW cavity modes. (A) Bright-field, transmission image of the cavity surrounded by the grating coupler; (B) high-frequency image of the structure, after the Fourier filtering by means of the beam-blocker on the collection path; (C) magnified image of the scattering pattern from the cavity centre. The central spot is selectively fed into the monochromator input slit, as sketched by the shadowed boxes; (D) scattering spectrum collected from the cavity centre. Two peaks appear at 584.5 nm and 597 nm, revealing corresponding cavity modes. Polarization-filtered spectra: (E) *x*-oriented polarization, (F) *y*-oriented polarization; (G) modulation of the peak intensities (background-subtracted) at 584.5 nm and 597 nm for different orientation angles *α* of the polarizer with respect to the *x*-axis.

A major issue in BSW-based devices is represented by the mode coupling with free-space radiation. This is often performed by means of bulky oil-immersion optics, gratings [24–26] and, more recently, with 3D printed refractive couplers [27] and individual Mie scatterers [28] In our case, a flat-optics element based on an annular grating inscribed into the DR1M-PPA layer well accomplishes this task, such that a white-light beam slightly focused by the microscope condenser -above the sample surface-can couple to BSW at different wavelengths (details in the Experimental section). Thanks to the grating shape, BSWs are focused toward the center of the structure [29], wherein denser periodic ring corrugations acting as a DBR surround a circular central region (here called the spacer). Such a resonator represents a type of two-dimensional implementation of a BSW cavity in a nanobeam [30]. In our case, BSW coupled from free-space are band-filtered by the DBR during the propagation on the 1DPC surface and possibly resonate within the inner spacer, at specific wavelength(s). In Figure 4A, a bright-field image of the cavity is presented (here called direct plane, DP, image). Due to the strong transmitted light, the coupling of BSWs and the interaction with the structure cannot be detected. Whit a beam-blocker placed on the objective BFP, implementing a high-pass Fourier filter on the angular spectrum of the collected light, the DP image in Figure 4B is obtained. Contributions to this image come substantially from leakage and scattered light related to the BSW excitation. For example, the bright annular region corresponding to the grating reveals the in/out-coupling of BSWs from/to free-space. More interestingly, a bright spot in the cavity center appears as due to the scattering of the cavity modes, after BSW have tunneled from the grating through the DBR. This bright spot is then precisely aligned to the input slit of the monochromator, for the spectral analysis (Figure 4C). The cavity spectrum is shown in Figure 4D, revealing a broad scattered background surmounted by two peaks. These two peaks are detected at *λ*_B_ = 584.5 nm and *λ*_R_ = 597 nm. After inserting a polarizer along the collection path, we found that the two peaks have maximum intensity corresponding to two orthogonal polarizations for the collected light (Figure 4E and F). As the orientation of the polarizer is varied, the intensities of the two peaks are complementarily modulated, as illustrated in Figure 4G. Specifically, the short-wavelength mode (*λ*_B_ = 584.5 nm) reaches the maximum of intensity when the transmissive axis of the polarizer is parallel to the *x*-axis (*α* = 0). Conversely, the long-wave mode (*λ*_R_ = 597 nm) has the maximum at *α* = π/2, indicating a polarization parallel to the *y*-axis.

**Figure 5:**
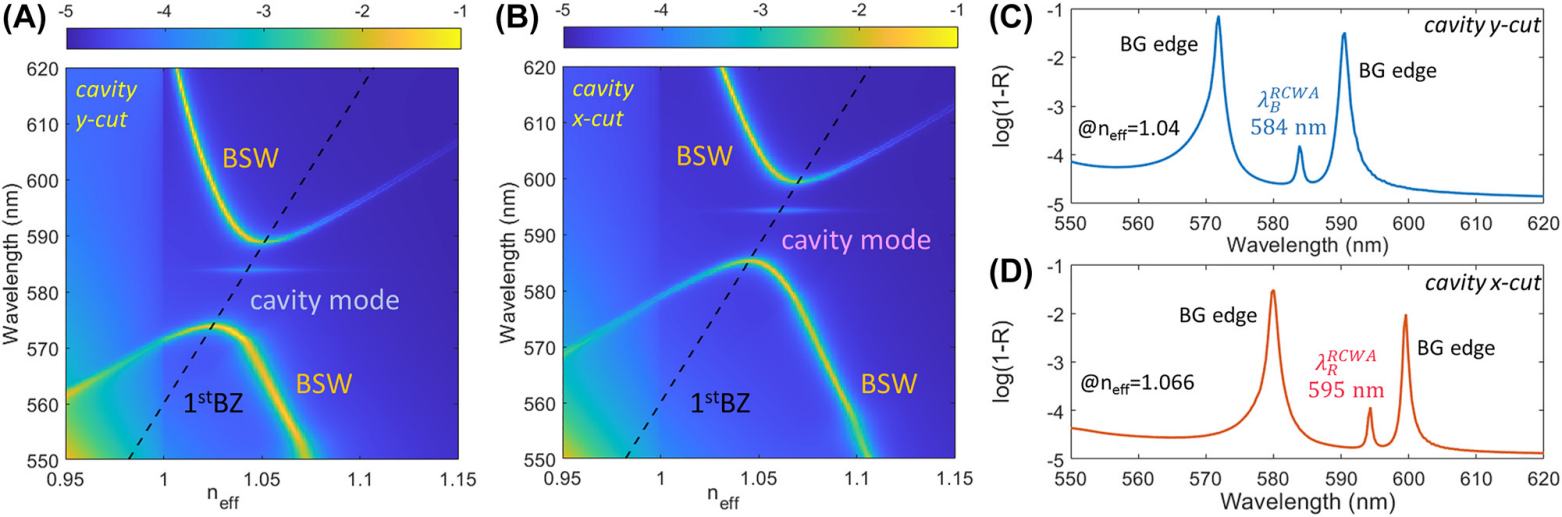
RCWA model of the cavity. Calculated angularly-resolved spectral maps log(1 − *R*) related to the cross-sectional cuts of the cavity along (A) the *y*-direction and (B) the *x*-direction. Calculations are performed assuming a TE-polarization, i.e. *x*-polarized in (A) and *y*-polarized in (B); black dashed line: boundary of the 1st Brillouin zone introduced by the DBR. The cavity modes are located in the BSW bandgap, as indicated; (C) and (D) linear plots of log(1 − *R*) for the *y*-cut at *n*_eff_ = 1.04 and for the *x*-cut at *n*_eff_ = 1.066, respectively.

The two substantially orthogonally polarized peaks are associated to resonant modes that are extremely sensitive to the cavity morphology. This can be understood with the help of a simplified model that is introduced here to investigate the spectral features of the cavity along the *x*- and *y*-directions separately (details on the computational model in the Experimental section). Accordingly, a periodically modulated film (DBR) with a central defect (spacer) surmounting the 1DPC is illuminated with a TE-polarized plane wave from the glass substrate at varying incident angles. The modelled DR1M-PPA structure also includes a uniform residual layer underlying the corrugation, with the central defect thicker than the surrounding DBR. Geometrical parameters for both the *x*- and *y*-profiles are defined in agreement with an analysis of the topography of the sample (Experimental section). From the calculated angularly-resolved spectral reflectivity *R*(*n*_eff_, *λ*), we plotted log(1 − *R*) in Figure 5A and B, to better visualize the dispersion of the folded BSW due to the DBR and the opening of a bandgap (BG) [31]. As expected from the Bragg law, the BSW folds about the boundary of the first Brillouin zone, given by $\lambda \left(n_{\text{eff}}\right)=2\cdot n_{\text{eff}}\cdot \Lambda_{\text{DBR}}$ . Being strongly bound to a surface, the BSW BG positions and widths are extremely sensitive to the DBR modulation depth and the residual layer thickness. Similarly to a Fabry–Perot resonator, defect (cavity) mode appears within the BG as a result of the presence of the spacer. In fact, two cavity modes are visible at $\lambda_{B}^{\text{RCWA}}=584\;\;nm$ (along the *y*-cut) and $\lambda_{R}^{\text{RCWA}}=595\;\;nm$ (along the *x*-cut), dispersionless in energy, with a broad spatial frequency spectrum (meaning spatial confinement in the corrugation direction). Worth to underline that, since the resonant modes are substantially TE-polarized, the cavity topography profile along the *x*-direction (*y*-direction) affects more strongly the mode that is *y*-polarized (*x*-polarized). Stated otherwise, the oscillating modes within the cavity have a transverse electric field, so that the mode at *λ*_*B*_ can be considered as mainly *x*-polarized, while the mode at *λ*_*R*_ is mainly *y*-polarized.

Despite the approximated model used, the peak position of the cavity modes are in good agreement with the experimental findings, the main reason for their spectral separation being the change of thickness in the residual DR1M-PPA layer beneath the corrugation. From Figure 5C and D, the high strength of the BG edge modes is expected to bring a significant contribution to the scattered radiation, as also found in the experimental observations at wavelengths above 580 nm (at shorter wavelengths, the observed scattering is very low, possibly because of a momentum mismatch between the BSW and the grating coupler, leading to BSW inefficiently launched toward the cavity). However, the use of the spatially-selective imaging system allowed us to partially alleviate the problem of the scattered background. The preferential collection of the light originating from the cavity centre, which is mainly due to the scattering of the cavity mode rather than the BG edge modes (distributed across the whole DBR) leads the cavity peaks to be clearly detected.

**Figure 6:**
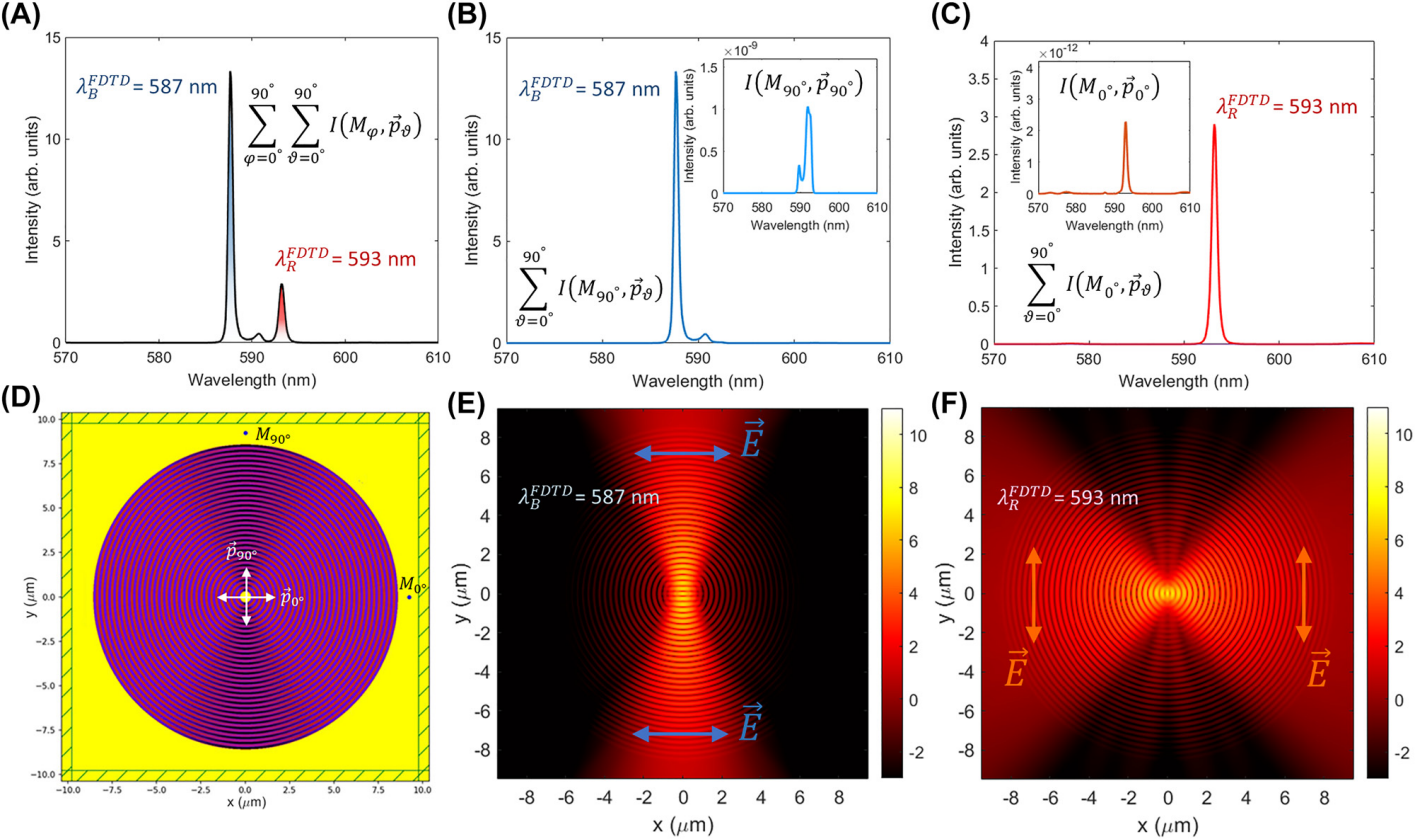
2D FDTD model of the BSW cavity. (A) Sum of intensity spectra collected by *M*_0°_ and *M*_90°_, produced by an incoherent superposition of sources ${\vec{p}}_{0{}^{\circ}}$ and ${\vec{p}}_{90{}^{\circ}}$ ; (B) intensity spectrum collected by *M*_90°_, produced by ${\vec{p}}_{0{}^{\circ}}$ and ${\vec{p}}_{90{}^{\circ}}$ incoherently superposed. Inset: spectrum at *M*_90°_, produced by ${\vec{p}}_{90{}^{\circ}}$ showing a negligible contribution to the mode coupling at $\lambda_{R}^{\text{FDTD}}$ ; (C) intensity spectrum collected by *M*_0°_, produced by ${\vec{p}}_{0{}^{\circ}}$ and ${\vec{p}}_{90{}^{\circ}}$ incoherently superposed. Inset: spectrum at *M*_0°_, produced by ${\vec{p}}_{0{}^{\circ}}$ showing a negligible contribution to the mode coupling at $\lambda_{R}^{\text{FDTD}}$ ; (D) geometry of the FDTD model with monitors *M*_0°_ and *M*_90°_ on the *x*-axis and *y*-axis, respectively. The two orthogonal sources are in the cavity centre as shown by white arrows; intensity distribution at $\lambda_{B}^{\text{FDTD}}=587\;\;nm$ (E) and $\lambda_{R}^{\text{FDTD}}=593\;\;nm$ (F). Both intensity distributions are calculated by the incoherent sum of the intensity distributions from the two source ${\vec{p}}_{0{}^{\circ}}$ and ${\vec{p}}_{90{}^{\circ}}$ . Colour scale is logarithmic. Arrows indicate the orientation of the electric field on the reference axes.

In order to get a deeper understanding of the polarization-sensitive splitting of the cavity mode, we built up a 2D finite difference time domain (FDTD) model based on the BSW effective refractive indexes [32]. The effective refractive index is modulated on the *xy*-plane according to the measured topography as described in the Experimental section. An emitting dipole is placed in the centre and oriented with its dipole momentum laying on the cavity plane, either parallel to the *x*-axis, ${\vec{p}}_{0{}^{\circ}}=\left(p,0\right)$ , or parallel to the *y*-axis, ${\vec{p}}_{90{}^{\circ}}=\left(0,p\right)$ . For each of the two dipole orientations, the intensity of the radiation leaking out of the cavity is detected by monitors *M*_φ_ (external to the DBR at φ = [0°, 90°]). Intensity *I* $\left(M_{\varphi },{\vec{p}}_{\vartheta }\right)=|E_{x}\left(x,y\right){|}^{2}+|E_{y}\left(x,y\right){|}^{2}$ refers to the fields detected at monitor *M*_φ_, emitted by source ${\vec{p}}_{\vartheta }$ . Recalling the TE-polarization of the BSW resonant modes, the intensities detected at monitors *M*_0°_ and *M*_90°_ are taken as representative of the intensity of light leaking out from the glass substrate and experimentally collected with the polarization oriented parallel the *y*-axis and *x*-axis, respectively. In Figure 6A, the sum of spectra detected by monitors *M*_0°_ and *M*_90°_, produced by an incoherent superposition of dipoles ${\vec{p}}_{0{}^{\circ}}$ and ${\vec{p}}_{90{}^{\circ}}$ , exhibits two peaks at $\lambda_{B}^{\text{FDTD}}=587\;\;nm$ and $\lambda_{R}^{\text{FDTD}}=593\;\;nm$ .

The two peaks correspond to the resonant modes of the anisotropic cavity. In fact, the intensity peak at $\lambda_{B}^{\text{FDTD}}$ is basically detected by monitor *M*_90°_ only (Figure 6B), thus indicating the *y*-axis as a preferential direction for the radiation power flowing out from the cavity. The polarization is substantially *x*-oriented. Conversely, the intensity peak at $\lambda_{R}^{\text{FDTD}}$ is detected by monitor *M*_0°_ only (Figure 6C), meaning that the power flow is preferentially along the *x*-direction, with a polarization substantially *y*-polarized. Worth to observe that the dipole orientations that couple more effectively to the cavity are ${\vec{p}}_{0{}^{\circ}}$ for the mode at $\lambda_{B}^{\text{FDTD}}$ and ${\vec{p}}_{90{}^{\circ}}$ for the mode at $\lambda_{R}^{\text{FDTD}}$ , respectively (see insets of Figure 6B and C). From the cavity geometry illustrated in Figure 6D, it is possible to appreciate the distribution in the effective refractive index modulation along the azimuthal direction and the related intensity distribution of the two resonant modes, shown in Figure 6E and F. After considering the electric field Cartesian components, we can conclude that the field is mainly *x*-polarized at $\lambda_{B}^{\text{FDTD}}$ and *y*-polarized at $\lambda_{R}^{\text{FDTD}}$ , which agrees well with the previous findings and the experimental observations.

### 2.3 Optical tuning of BSW cavity resonances

**Figure 7:**
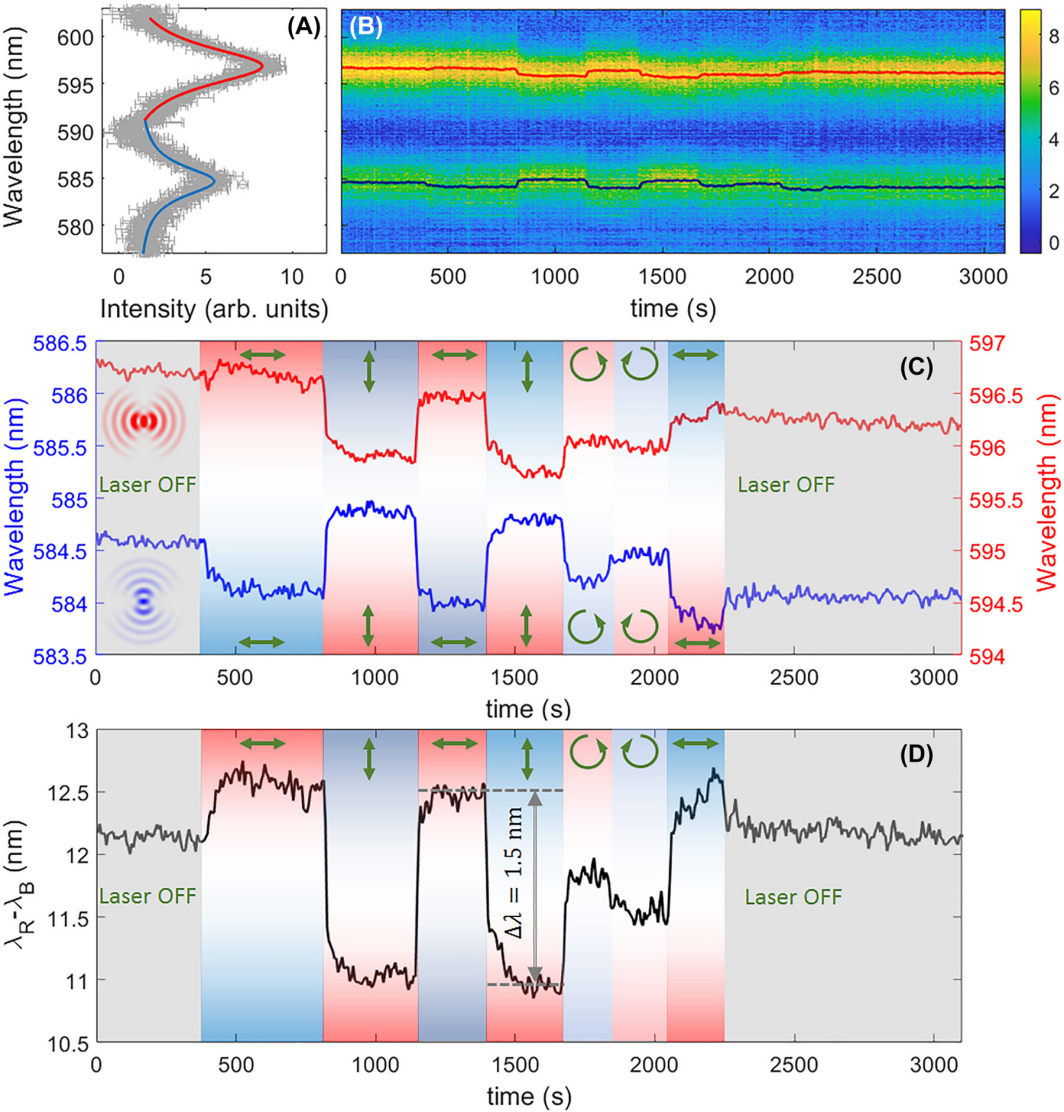
Spectral shift of the BSW cavity modes. (A) Background-subtracted, measured spectral profile of the intensity peaks associated to the *x*-polarized mode at *λ*_*B*_  *≅* 584.5 nm and the *y*-polarized mode at *λ*_*R*_  *≅* 597 nm. Lorentzian fits are also shown; (B) time evolution of the collected scattering spectra during laser irradiation; (C) time evolution of the central wavelengths *λ*_*B*_ and *λ*_*R*_ obtained after fitting. Green arrows indicate the polarization state of the illuminating laser: *y*-oriented (vertical), *x*-oriented (horizontal), left- or right-handed; (D) time evolution of the relative spectral shift of the orthogonal cavity modes. A tuning over about 1.5 nm spectral range is demonstrated.

The two cavity modes with substantially orthogonal polarizations and spatial distributions are suitable for probing the in-plane anisotropy induced on the DR1M-PPA structure. After background subtraction, the cavity modes are spectrally observed as shown in Figure 7A. In Figure 7B the time evolution of the spectral position of the cavity modes is illustrated, while the cavity is illuminated with the laser beam in different polarization states (laser power 0.15 mW, laser spot diameter 80 μm). In order to monitor both peaks, no polarization filters have been inserted along the illumination and the collection paths. Figure 7C shows a more detailed view of the mode peak positions over time. The opposite spectral shifts of the two modes are evident and can be explained by invoking the mechanism underlying the formation of optical anisotropy previously illustrated. As the laser is linearly polarized along the *x*-direction, the *x*-polarized mode peak at *λ*_B_ blue-shifts because of a smaller extraordinary refractive index n_e_ induced in that direction. Conversely, the *y*-polarized mode at *λ*_R_ slightly red-shifts because more DR1M dipoles tend to be oriented in the *y*- and *z*-direction. The difference in the spectral shift amplitude observed for the two modes can be explained by invoking photo-thermal effects, whereby a substantial (isotropic) decrease of the refractive index is produced as the polymer heats up. The *λ*_R_ mode senses a net change of refractive index in the *y*-direction given by the induced birefringence and heating, wherein the two effects have opposite signs. For the *λ*_B_ mode, both birefringence and heating produce a negative change of refractive index along the *x*-direction, resulting in a significant blue-shift. After the sample is brought to thermal equilibrium, the spectral shifts of the BSW modes are mirrored for the two orthogonal modes, as it is possible to observe each time the polarization of the laser illumination is rotated by 90°.

Thanks to the capability of the DR1M-PPA compound to sense the polarization state of the illuminating radiation [33, 34], we detected some asymmetry in the peak shift when a circularly polarized light with opposite handedness is used. This suggests that the laser polarization is likely to be slightly elliptical. When the laser is switched off, the optical anisotropy induced in the DR1M is kept as long as the thermal relaxation process allows it. By looking the relative spectral shift (*λ*_R_ − *λ*_B_) an overall tuning of Δ*λ* = 1.5 nm between the two modes is found (Figure 7D), with a rather accurate recovery of the initial condition after the laser is switched off. The optical anisotropy here is induced only on a very small area, contrary to the case of BSW propagating on the flat 1DPC with a homogeneous DR1M-PPA coating, where the laser spot covered the full field of view of the collecting objective (because of the BFP-based detection).

## 3 Conclusions

We presented a hybrid nanophotonic device combining a dielectric photonic crystal made of amorphous materials with a light-responsive flat-optics structure based onto a functionalized polymeric blend. In particular, a resonant cavity for BSWs is nanofabricated into a thin azobenzene-containing polymer layer deposited on top of a high-quality 1DPC via thermal-scanning probe lithography. The photo-switching of the azobenzenes is exploited not only to produce an overall reversible change of the refractive index, but to optically inscribe an optical anisotropy. Differently from other methods exploiting e.g. thermo-optical, mechanical or electro-optical effects [35], the all-optical control allows a precise selection of very localized regions (such as tiny recesses), which makes this approach very attractive for densely packed devices.

By leveraging the ability of the polymeric structure to produce in-plane anisotropy after proper laser irradiation, we demonstrate the possibility to perform a complex manipulation of the response of resonant structures for surface waves, provided the cavity modes are TE-polarized, as in the case of BSWs. The cavity itself exhibits topographic nanofeatures that make it resonating according to two orthogonally polarized modes, at different energies. The optically-controlled anisotropy of the azobenzenes allows the spectral positions of the resonant modes to be tuned in several ways. For example, an illumination with a circularly polarized laser causes an isotropic decrease of the in-plane refractive index components, so that both modes can be simultaneously blue-shifted, starting from a configuration of zero birefringence. Moreover, the same illumination with a circular polarization can produce a red-shift on one mode and a blue-shift on the other, in case initial in-plane anisotropy is already inscribed into the polymer. When a linearly polarized laser is used, a strong decrease of refractive index in the polarization direction is observed, thus resulting in a blue-shift of the cavity mode polarized in that direction and a typical red-shift of the orthogonal mode. In conclusion, by using moderate laser power densities, we have shown that the cavity resonance wavelengths can be easily shifted over a range of about 1.5 nm, with time constants of about 10–20 s. Potential improvements of this result are expected in case more efficient light-responsive units capable of larger optically-induced birefringence are employed [36].

In a more general perspective, the integration of a light-responsive layer within dielectric photonic structures has been shown to provide both an additional degree of freedom for surface patterning and an optically addressable mean for controlling birefringence. These advantages are gathered without being significantly detrimental toward the optical response of the structure underneath. For example, the quality of the surface modes here considered is still rather high, with resonance widths much smaller than many plasmonic counterparts, because of the lower losses. We anticipate that applications of this approach can be particularly foreseen in emitting photonic devices [37] hosting extended [38–41] or single sources [42–47], wherein the photonic modes can be finely adjusted in polarization and energy to match the emitter orientation [48, 49] and wavelength, generally aiming at a coupling optimization.

## 4 Experimental section

### 4.1 Photonic structure

The one-dimensional photonic crystal (1DPC) sustaining TE-polarized BSW is a stack of Ta_2_O_5_ and SiO_2_ layers on a glass coverslip. The stack sequence is [Ta_2_O_5_–SiO_2_] × 6–Ta_2_O_5_–SiO_2_. The Ta_2_O_5_ layer (refractive index 2.08) is 95 nm thick, the SiO_2_ layer (refractive index 1.46) is 137 nm thick. The last SiO_2_ layer of the stack has a reduced thickness of 127 nm thick. On top of the stack, a 65 ± 3 nm thick polymeric layer is spun. The polymeric film is obtained from a mixture of polyphthalaldehyde (PPA, Phoenix 81 from Allresist) and disperse red 1 methacrylate (DR1M, from Sigma-Aldrich), a widely used compound containing azobenzene groups. The DR1M-PPA film has a twofold function: (i) it enables the nanopatterning of the 1DPC surface with the grating coupler and the cavity, without introducing dramatic perturbations to the sustained BSWs, (ii) it provides an optically active medium whose anisotropy can be light-controlled via the external laser radiation. It is well known that pre-fabricated azopolymeric structures can undergo significant topographical changes when irradiated at energies promoting the cyclic photoisomerization of azobenzenes [50, 51]. Related effects such as directional photofluidization [52] and mass migration [53], which are involved in the formation of surface relief gratings [54], have been shown to cause severe loss of integrity in pre-fabricated micro and nano-structures [55]. Therefore, in order to mitigate these effects and improve the mechanical stability of the pattern under a prolonged laser irradiation, we use a DR1M-PPA blend (weight ratio 30%), which offers the additional advantage of allowing patterning by means of a probe-assisted heating technique thanks to the high sensitivity to local heating promoted by the PPA component [56]. In this way, the direct nanopatterning of the DR1M-PPA film has been possible by means of a novel thermal-scanning probe lithography (t-SPL) that is able to control the local sublimation of the polymer layer thanks to the action of a sharp silicon tip providing temperature and time controlled heat pulses (Nanofrazor Scholar system, Heidelberg Instruments) [13]. By exploiting this nanolithographic technique, all dielectric photonic cavities can be nanopatterned at the surface of functionalized polymer layers, achieving high spatial resolution at the subwavelength scale.

The PPA-DR1M layer is about 65 nm thick. This permits to obtain: (i) a BSW dispersion spectrally far enough from the DR1M absorption maximum; (ii) a good spatial resolution of the patterned features in t-SPL. In fact, for thinner films we observed a strong damping of the BSW resonant mode due to absorption losses, while for thicker films the quality of the lithographed structures was increasingly deteriorating.

**Figure 8:**
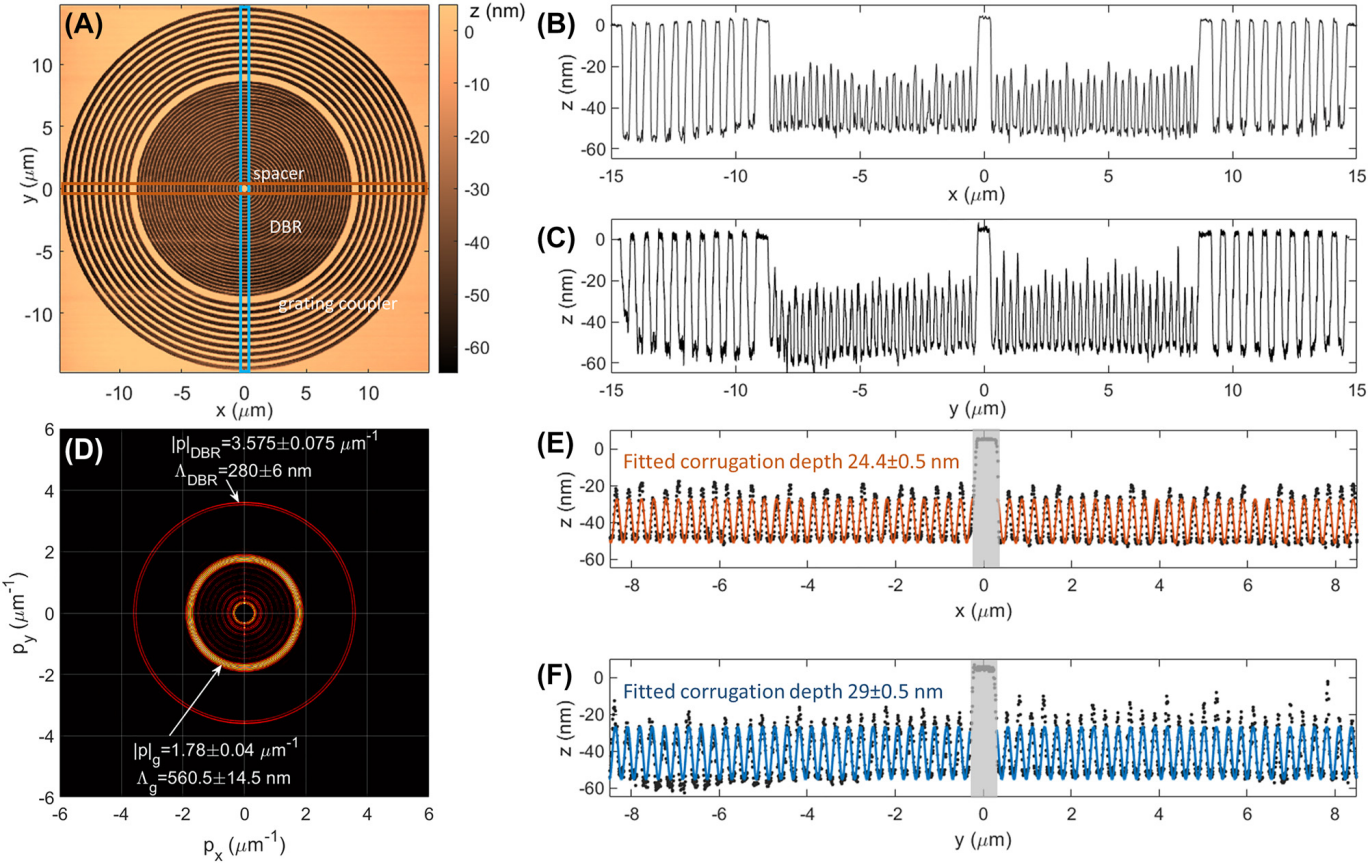
Topography of the DR1M-PPA structure. (A) Topography map. Blue and red boxes define the integration regions for plotting the cross sections; (B) horizontal (*x*-direction) and (C) vertical (*y*-direction) cross sections of the pattern; (D) modulus of the Fourier transform (FFT) calculated from the topography map. The zero-order is masked to zero. The two modulations related to DBR and grating coupler are indicated together with the estimated periods; experimental data and fitted profiles of the DBR corrugation along (E) the *x*-direction and (F) the *y*-direction. The fitting curve is a single-frequency harmonic term. The modulation depths estimated after the fit are indicated for both orthogonal profiles.

Figure 8A, shows the topographic image map acquired *in-situ* by the Nanofrazor system, exploiting the same tip to scan the patterned region in contact mode. The *xy* axis indicated in the figure defines the reference system used throughout this work. In Figure 8B and C, the horizontal (*x*-direction) and vertical (*y*-direction) profiles of the structure topography are shown. It is possible to appreciate lower modulation amplitude of the DBR as compared to the outer grating coupler, which spans across almost the total film thickness (≈65 nm). A two-dimensional fast Fourier transform (FFT2) of the topography map is calculated (Figure 8D), revealing two bright rings associated to the periods of the grating coupler, Λ_*g*_ and the DBR, Λ_DBR_. After the FFT2-based spatial frequency analysis, we estimate Λ_*g*_ = 560.5 ± 15.5 nm and Λ_DBR_ = 280 ± 6 nm for the grating coupler and the DBR, respectively, which are well matching the design parameters. Worth to note that the FFT amplitude of the frequency term related to the DBR exhibits a slight anisotropy, suggesting that the modulation depth of the DBR may vary with the azimuthal angle, over the plane.

We carefully fitted the DBR topographic profiles along the *x*- and *y*-directions by using a single-frequency harmonic function given by a linear combination of a sine and a cosine (in addition to a baseline, constant term). Results are shown in Figure 8E and F. In the fitting procedure, the inner spacer has been excluded (shadowed region) and the profile portions beforehand and afterwards the spacer have been fitted separately. The average corrugation depths obtained from the fit along the *x*- and *y*-directions are *h*_*x*_ = 24.4 ± 0.5 nm and *h*_*y*_ = 29 ± 0.5 nm, respectively. Although very small, the depth modulation difference in the DBR along the two orthogonal directions is not negligible as it introduces a detectable optical anisotropy of the cavity, causing a splitting of the BSW resonances into two orthogonally polarized modes at different energies.

### 4.2 Experimental setup

Optical measurements are performed on a home-made setup based on a modified inverted microscope (Nikon Ti2-E), as sketched in Supplementary Figure S1. The sample is mounted face-up onto a planar sample holder. An oil-immersion objective (Nikon, NA = 1.49) is contacted to the bottom side of the thin glass coverslip (substrate) hosting the photonic structure. White light illumination is provided by a halogen lamp along two alternative paths: (i) a transmission path, whereby light is incident from above the sample, after being slightly focused by the microscope condenser (maximum NA = 0.3); (ii) a reflection path, whereby light is incident from the bottom, after being focused by the high-NA objective. The Gaussian laser beam (CW Torus532 from Novanta Photonics, former Laser Quantum) impinges on the sample from the top, along the transmission path, after being expanded and polarization-controlled separately from the white light. The laser is slightly focused onto the sample by the condenser. When cavities are considered, the laser spot has a diameter of about 50 μm, instead, when the flat 1DPC is considered, the spot diameter is about 2.5 mm.

BSW dispersion measurements are performed in back focal plane imaging mode [57], with the illumination light provided through the reflection path. We highlight that the reflectivity dip associated to the coupling of BSW cannot be directly detected on the BFP when the dispersive grating is set to image the 0th diffraction order on the camera. In fact, in this configuration, reflected intensities at all wavelength sum up incoherently on the camera plane, for any given *n*_eff_ value. Spectral measurements on the cavities are performed in the direct plane (DP) imaging configuration, by aligning the cavity center with the entrance slit.

### 4.3 Computational modelling

The angular-dependent spectral reflectivity map of the planar 1DPC is calculated by means of a MATLAB implementation of the transfer matrix method. The illumination is assumed as a TE-polarized plane wave incident on the 1DPC from the glass substrate at specific angles and wavelengths.

As the surface pattern is introduced, we neglect the presence of the grating coupler and consider the resonant cavity only (i.e. the DBR and the inner spacer), leaving the design of the grating as a mere application of the Bragg law. Two approximate methods have been adopted. In a first approach, the freely available MATLAB package implementing the rigorous coupled waves analysis (RCWA) RETICOLO [58, 59] is used to model the full multilayer and the cavity on top, assumed as a one-dimensional (1D) corrugation with a defect spacer, along the two orthogonal *x*- and *y*-direction (see Supplementary Figure S2 and related description). In a second approach, a two-dimensional finite difference time domain (FDTD) model [60] based on the effective index (EI) approximation [61, 62] is used to compute the spectral response of the circular cavity only, without explicitly keeping into account the multilayer beneath and the outer annular grating. The EI approach has been demonstrated useful to accomplish design tasks for complex BSW platforms [63]. Details on the EI distribution based on the cavity topography are illustrated in the Supplementary Figure S3 and related description.

Although these models cannot recapitulate precisely the optical response of the three-dimensional structure, they offer an acceptable compromise between accuracy and running time, effective enough for the structure design and the interpretation of the experimental data.

## Supplementary Material

Supplementary Material Details
