# Supplementary material for: Spectral tuning of Bloch Surface Wave resonances by light-controlled optical anisotropy
Source: Nanophotonics. 2023 Feb 20;12(6):1091–104. doi: 10.1515/nanoph-2022-0609 (PMC11501718; doi:10.1515/nanoph-2022-0609)
Supplement: Supplementary file 1 — Supplementary Material Details [file j_nanoph-2022-0609_suppl.pdf]

Supplementary Materials

Spectral tuning of Bloch Surface Wave resonances by light-controlled optical anisotropy

Niccolò Marcucci[§], Maria Caterina Giordano[§], Giorgio Zambito, Adriano Troia, Francesco Buatier de Mongeot, and Emiliano Descrovi

Corresponding Author
emiliano.descrovi@polito.it

Authors

Niccolò Marcucci – Dipartimento di Scienza Applicata e Tecnologia, Politecnico di Torino, Corso Duca degli Abruzzi 24, Torino, 10129, Italy.

Maria Caterina Giordano – Dipartimento di Fisica, Università di Genova, Via Dodecaneso 33, Genova, 16146, Italy.

Giorgio Zambito - Dipartimento di Fisica, Università di Genova, Via Dodecaneso 33, Genova, 16146, Italy.

Adriano Troia – Istituto Nazionale di Ricerca Metrologica (INRiM), Strada delle Cacce 91, Torino 10135, Italy

Francesco Buatier de Mongeot - Dipartimento di Fisica, Università di Genova, Via Dodecaneso 33, Genova, 16146, Italy.

Emiliano Descrovi - Dipartimento di Scienza Applicata e Tecnologia, Politecnico di Torino, Corso Duca degli Abruzzi 24, Torino, 10129, Italy.

Author contributions

[§] These authors contributed equally to this work.

Experimental setup

Optical measurements are performed on a home-made setup based on a modified inverted microscope (Nikon Ti2-E), as sketched in supplementary Figure S1. The sample is mounted face-up onto a planar sample holder. An oil-immersion objective (Nikon, NA=1.49) is contacted to the bottom side of the thin glass coverslip (substrate) hosting the photonic structure. White light illumination is provided by a halogen lamp along two alternative paths: (i) a transmission path, whereby light is incident from above the sample, after being slightly focused by the microscope condenser (maximum NA=0.3); (ii) a reflection path, whereby light is incident from the bottom, after being focused by the high-NA objective. The Gaussian laser beam (CW Torus532 from Novanta Photonics, former Laser Quantum) impinges on the sample from the top, along the transmission path, after being expanded and polarization-controlled separately from the white light. The laser is slightly focused onto the sample by the condenser. When cavities are considered, the laser spot has a diameter of about 50 μm, instead, when the flat 1DPC is considered, the spot diameter is about 2.5 mm.

The collected light (white light and laser) is spectrally filtered by a long-pass filter (Thorlabs FELH0550) and an image of the sample is formed at the lateral output port of the microscope. A lens L1 produces the Fourier Transform of the image on its focal plane (here called the Back Focal Plane -BFP) and possibly polarization-filtered by means of a polarizer. On the BFP, Fourier filtering elements such as pinholes or beam blockers can be inserted. Further on, light passes through an imaging box adapted to operate in two alternative imaging modes. In the Direct Plane (DP) Imaging, an additional Fourier Transformation is performed by lens L2, so that the image of the sample formed at the lateral output port of the microscope is projected onto the entrance slit of a 300 mm focal-length monochromator (Acton SpectraPro-300i). A beam blocker placed on the BFP allows the transmitted zero-order background to be filtered out, leaving only the higher frequencies of the angular spectrum to contribute to the image. In the Back Focal Plane (BFP) imaging, the intensity distribution formed onto the BFP plane is projected onto the monochromator entrance slit by means of the lens L3, in a 2f-2f configuration. The slit of the monochromator can be opened/closed so that specific regions of

the DP or the BFP can be selectively analyzed spectrally. The monochromator mounts three dispersive gratings: with 1200 lines/mm, 300 lines/mm and 150/lines/mm. The detection is performed with a monochromatic CMOS camera (Thorlabs CS505MU1) at the output port of the monochromator.

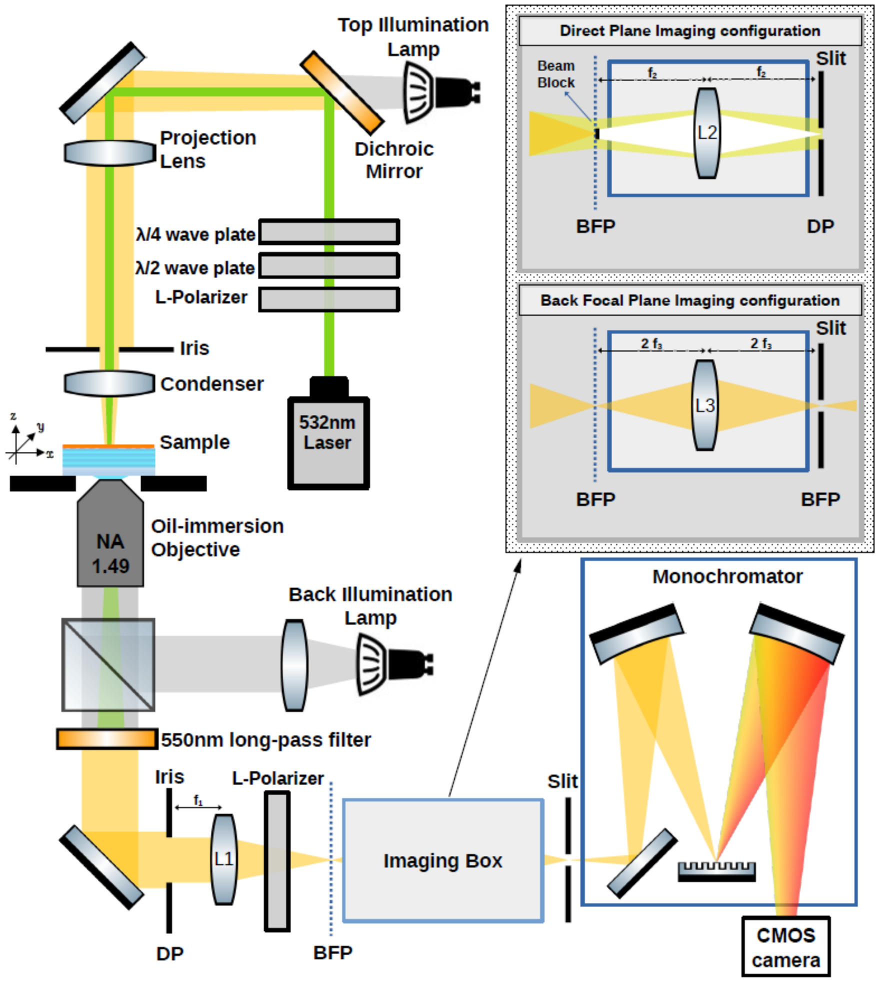

Figure S1. Optical setup. Based on an inverted microscope mounting an oil-immersion objective, the setup allows a white-light illumination either from above (the air side) or from below (the glass substrate side) the sample. The control laser used to produce the desired birefringence in the DR1M-PPA film is incident onto the sample surface from the top, after being slightly focused by the microscope condenser. Measurements are performed in two configurations: (i) the Direct Plane (DP) imaging or (ii) the Back Focal Plane (BFP) imaging. A dispersive spectrometer allows for spectral measurements on selected areas of the collected images in both imaging configurations.

Computational modelling

In a first approach, the freely available MATLAB package implementing the Rigorous Coupled Waves Analysis (RCWA) RETICOLO is used to model the full multilayer and the cavity on top,

assumed as a one-dimensional (1D) corrugation with a defect spacer, along the two orthogonal x- and y- direction. Being based on a Fourier decomposition of the refractive index profile, RCWA assumes the modeled structure to fit within a calculation domain (a cell) that is periodically repeated along one direction. This poses serious issues in case a single cavity is considered (i.e. a periodically corrugated profile with a localized defect). As a viable solution, we set the calculation domain as the super-cell shown in Figure S2, where the inner spacer is surrounded by a very large number of DBR periods. In this way, we attempt to reduce the coupling between horizontally adjacent super-cells (and cavities). Obviously, as the number of DBR periods becomes larger, the number of Fourier harmonics components needed to be retained in the calculation grows as well, thus posing unavoidable limitations to the calculation accuracy.

In Figure S2A a sketch of the geometry used in the RCWA is shown. The model addresses the cavity only, without the coupling grating. Illumination is a TE-polarized plane wave propagating from the glass substrates at different incidence angles and wavelengths. The DR1M-PPA structure on top of the 1DPC is constituted by a residual uniform layer (thickness t_{res}), a modulated DBR region (thickness t_{DBR}) and a spacer region having an additional thickness of $t_{spacer.}$ From the AFM analysis, $t_{res} + t_{DBR} + t_{spacer} = 65$ nm. In order to address the slight anisotropy of the cavity, as highlighted from the analysis of the topography, two different cases are considered, referring to cross-sectional cuts of the structure along either the x- or the y-direction. In particular, along the x-direction, $t_{res} = 15$ nm and $t_{DBR} = 24$ nm, while along the y-direction, $t_{res} = 2$ nm and $t_{DBR} = 29$ nm. For both directions, the DBR period is $\Lambda_{DBR} = 280$ nm, the spacer width is $W_{spacer} = 560$ nm and the fill factor is 0.5. A complex refractive index is given to the DR1M-PPA, $n_p = 1.615 + i1 \cdot 10^{-3}$ in order to generally take into account absorption losses by the azodye. In the results presented here, we consider a cavity with N_{DBR}=350 at each side of the inner spacer, with N_F=1200 Fourier terms. These values have been chosen after convergence tests. In the exemplary Figure S2B,C reflectivity spectra of the cavity (x-cut) are calculated for n_{eff}=1.066 as a function of N_{DBR} (keeping N_f=1200). Moreover,

for 800<N_F<2000 the spectral position of the cavity mode and the corresponding reflectivity value fluctuate by 0.2 nm and 0.01 respectively.

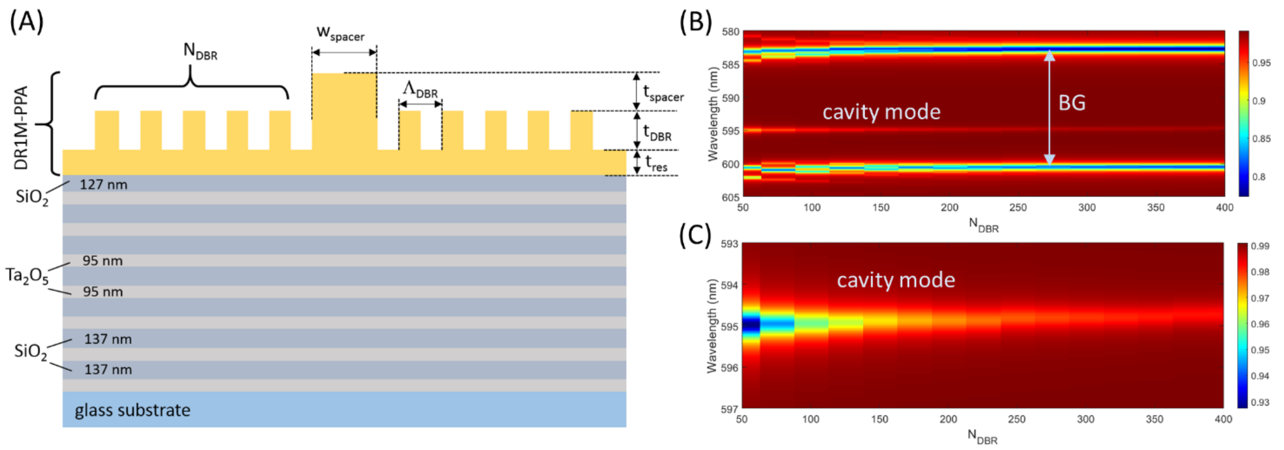

Figure S2. RCWA model. (A) Geometry of the super-cell including the 1DPC and the 1D cavity on top; (B,C) reflectivity spectra calculated at different N_{DBR}, keeping N_F=1200. The fluctuations of the cavity mode position is about 0.2 nm for N_{DBR}>200.

In a second approach, a two-dimensional Finite Difference Time domain (FDTD) model based on the effective index approximation is used to compute the spectral response of the circular cavity only, without explicitly keeping into account the multilayer beneath and the outer annular grating.

According to this 2D approximated model, each relief with a given thickness has an associated effective refractive index defined by the BSW dispersion on a flat 1DPC loaded with a uniform DR1M-PPA film with the same thickness. It is thus assumed that the effective refractive index of the reliefs is not affected by the lateral size of the reliefs themselves. In the present case, the effective refractive dispersion is modelled according to the structure topography, in order to take into account the anisotropy along the azimuthal angle φ on the xy-plane. The experimental DBR modulation and the residual layer thickness extracted from the AFM analysis are shown in Figure S3A together with the DBR modulation profile $t_{DBR}(\varphi)$ and the residual layer profile $t_{res}(\varphi)$ employed in the FDTD mode. In addition, in order to account for the different mode volume of the cavity modes as compared to the 1D RCWA model, the spacer width is set as $W_{spacer} = 640$ nm, which is consistent with the

measured profile as shown in Figure S3B. Accordingly, the resulting cavity profile is $z_{caviy}(r,\varphi)$ distribution, in polar coordinates $\begin{cases} r = \sqrt{x^2+y^2} \\ \varphi = \mathrm{tg}^{-1}\left(\frac{y}{x}\right) \end{cases}$ is then:

$$z_{caviy}(r,\varphi) = \begin{cases} \frac{A+B}{2} + \left(\frac{A-B}{2}\right) \cdot \mathrm{sgn}\left[\cos\left(K_{DBR}\left(r - \frac{1}{2}\mathrm{W_{spacer}}\right)\right)\right] & \text{if } r - \frac{1}{2}\mathrm{W_{spacer}} > 0 \\ C & \text{if } r - \frac{1}{2}\mathrm{W_{spacer}} \leq 0 \end{cases}$$

whit $K_{DBR} = 2\pi\Lambda_{DBR}^{-1}$; $A(\varphi) = \left(t_{DBR}^{max} + t_{res}^{min}\right) + \left(t_{res}^{max} - t_{res}^{min} + t_{DBR}^{min} - t_{DBR}^{max}\right) \cdot (1 - \sin^8\varphi)$; $B(\varphi) = t_{res}^{min} + \left(t_{res}^{max} - t_{res}^{min}\right) \cdot (1 - \sin^8\varphi)$; $C = 65$ nm (Figure S3C). In Figure S3D, the modelled topographic profiles along the x- and y-direction are presented as well. Cavity modes are excited by inserting an emitting dipole in the spacer centre, with momentum orientation either parallel to the x-axis $\vec{p}_{0°} = (p,0)$ or parallel to the y-axis $\vec{p}_{90°} = (0,p)$ and calculating the intensity of the radiation flowing out of the cavity, as detected by monitors M_φ at φ=[0°, 90°] outside the DBR. Intensity $I(M_\varphi, \vec{p}_\vartheta)$ indicates the intensity detected at monitor M_φ emitted by the source $\vec{p}_\vartheta$.

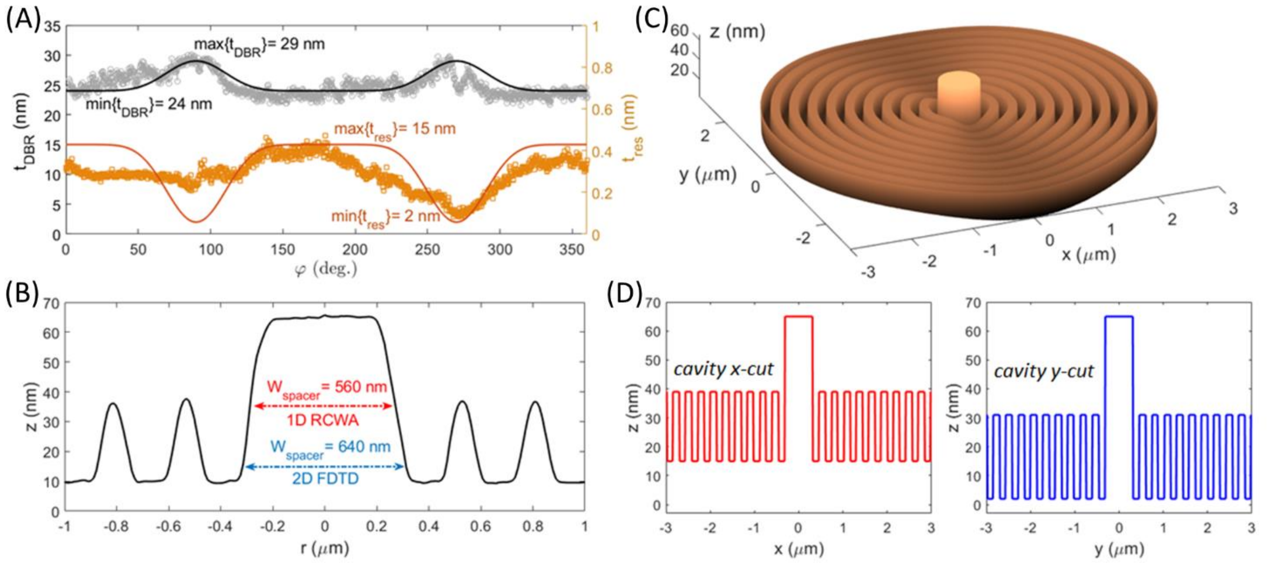

Figure S3. 2D FDTD model. (A) Azimuthal profile of the DBR modulation thickness (gray circles) and the residual layer thickness (orange squares) from the AFM analysis together with the analytical profile $t_{DBR}(\varphi)$ (black solid line) and $t_{res}(\varphi)$ (orange solid line) used in the FDTD model; (B) topographic radial profile of the cavity spacer, averaged over φ. For clarity of illustration, only few DBR periods are shown in the image, whilst in FDTD calculations, the number of DBR periods is set to N_{DBR}=30; (C) surface rendering of the cavity topography in the FDTD model; (D) cross-sectional topographic profiles of the cavity along the x- and y-cut.
